# Heterogeneity Analysis and Diagnosis of Complex Diseases Based on Deep Learning Method

**DOI:** 10.1038/s41598-018-24588-5

**Published:** 2018-04-18

**Authors:** Xiong Li, Liyue Liu, Juan Zhou, Che Wang

**Affiliations:** 1 School of Software, East China Jiaotong University, Nanchang, Jiangxi 330013 China; 2 School of Electrical and Automation Engineering, East China Jiaotong University, Nanchang, Jiangxi 330013 China

## Abstract

Understanding genetic mechanism of complex diseases is a serious challenge. Existing methods often neglect the heterogeneity phenomenon of complex diseases, resulting in lack of power or low reproducibility. Addressing heterogeneity when detecting epistatic single nucleotide polymorphisms (SNPs) can enhance the power of association studies and improve prediction performance of complex diseases diagnosis. In this study, we propose a three-stage framework including epistasis detection, clustering and prediction to address both epistasis and heterogeneity of complex diseases based on deep learning method. The epistasis detection stage applies a multi-objective optimization method to find several candidate sets of epistatic SNPs which contribute to different subtypes of complex diseases. Then, a K-means clustering algorithm is used to define subtypes of the case group. Finally, a deep learning model has been trained for disease prediction based on graphics processing unit (GPU). Experimental results on pure and heterogeneous datasets show that our method has potential practicality and can serve as a possible alternative to other methods. Therefore, when epistasis and heterogeneity exist at the same time, our method is especially suitable for diagnosis of complex diseases.

## Introduction

Since complex diseases such as cancer, diabetes and so on pose a very big threat to human health, they have been extensively studied in the past decades^1^. However, the underlying pathogenesis of complex diseases is still not clearly known. With the rapid development of genomics technologies, the big data of variations on DNA level such as SNP and CNV (copy number variation) allow comprehensive characterization of complex diseases and provide potential biomarkers to predict the status of complex diseases.

Due to the ‘missing heritability’ and lack of reproducibility, the exploration of relationships between SNPs and complex diseases have been transferred from single variation to biomarkers interactions which are defined as epistasis^2^. Epistasis analysis on genome-wide faces at least three challenges. First, as the number of variants increases, the combination space expands exponentially, resulting in the ‘curse of dimensionality’ problem. Furthermore, when the higher order of epistasis is considered, the situation becomes even worse. Second, numerous biomarkers epistasis will be tested for significant association with complex diseases from statistical view, leading to the ‘multiple testing’ problem. Therefore, the association results may be false positive and are hard to be replicated. Third, from the statistical learning view, the large number of SNPs but small sample poses the ‘high dimensional and small sample size’ problem, which causes the lack of generalization ability.

By now, lots of methods have been proposed to analyze the epistasis and can be roughly classified into exhaustive method^3,4^, heuristic method^5,6^ and machine learning method^7,8^. When handling the large number of loci, exhaustive methods take huge computational costs. There are many strategies to accelerate process of exhaustive search. For example, multifactor dimensionality reduction (MDR)^3^ and exhaustive search based on multi-objective optimization (ESMO)^4^ apply parallel computing to save running time. With using the exhaustive strategy, all of epistatic combinations have been tested, so that the power of association studies is relatively higher. Heuristic methods such as AntEpiSeeker^9^ and MACOED^10^ use prior knowledge or information retrieved by swarm intelligence to narrow down the combination space. The main limitation of heuristic methods is randomness. It means that the results may be different during different iterations. Machine learning based methods such as logistic regression^11^ and Bayesian network^12^ operate as a black box which indirectly profile the relationship between genetic variants and complex diseases.

In addition to epistasis, heterogeneity is another key factor contributing to complexity of locating the pathogenesis loci of complex diseases^13^. Due to heterogeneity, there may be several different combination patterns of epistasis existing in the cases. And, different patterns contribute to different subtypes of complex disease. In some situations, the subtypes may be caused by incorrectly sampled or classified, so that data stratification is a common way to preprocess the data^14^. However, if the subtypes of complex diseases really exist, data stratification could lead to the loss of power. In this work, we assume that all the samples are well defined and sampled and heterogeneity analysis is considered as potential pathogenic pattern recognition and multiple classification.

As far as we know, only a few approaches can concurrently consider both epistasis and heterogeneity in association studies without resorting to some forms of stratification. For instance, ESMO not only applies multiple scoring criteria to complementarily evaluating each candidate epistatic combination, but also returns multiple epistatic combinations corresponding to different subtypes. MDR profiles heterogeneity by ranking multiple epistatic models according to the prediction accuracy. However, the prediction accuracy of these methods still needs to be improved. More importantly, MDR only classified samples into two categories: case and control, without considering multiple subtypes, namely multi-classification.

**Figure 1:**
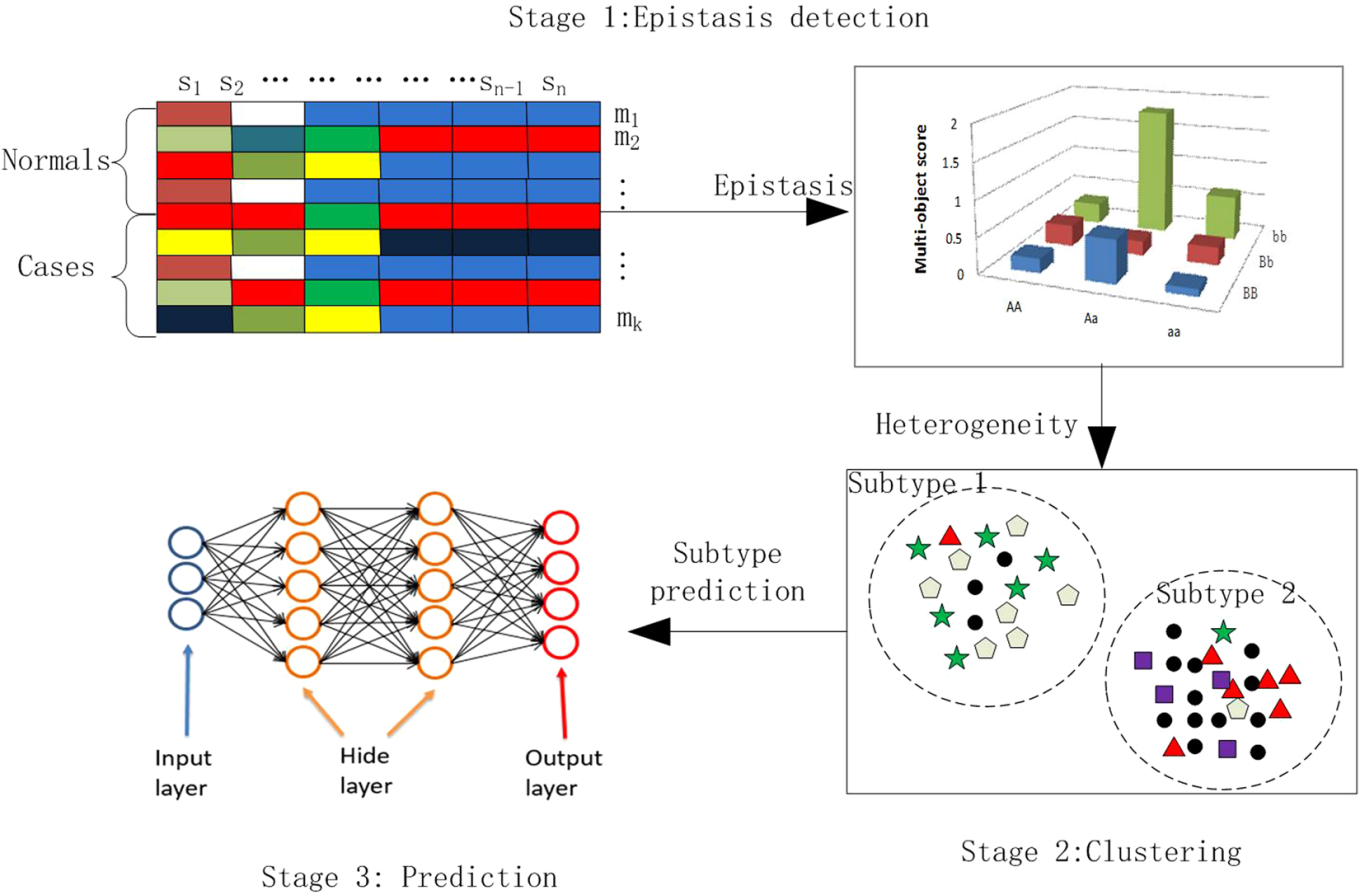
The three-stage of the DPEH.

In this study, we propose a deep learning method for epistasis and heterogeneity analysis (DPEH). DPEH detects epistasis and heterogeneity with using a three-stage framework as depicted in Fig. 1. After introducing the method of DPEH, the experimental results both on pure and heterogeneous datasets are provided to demonstrate the practicality of DPEH.

## Results

**Figure 2:**
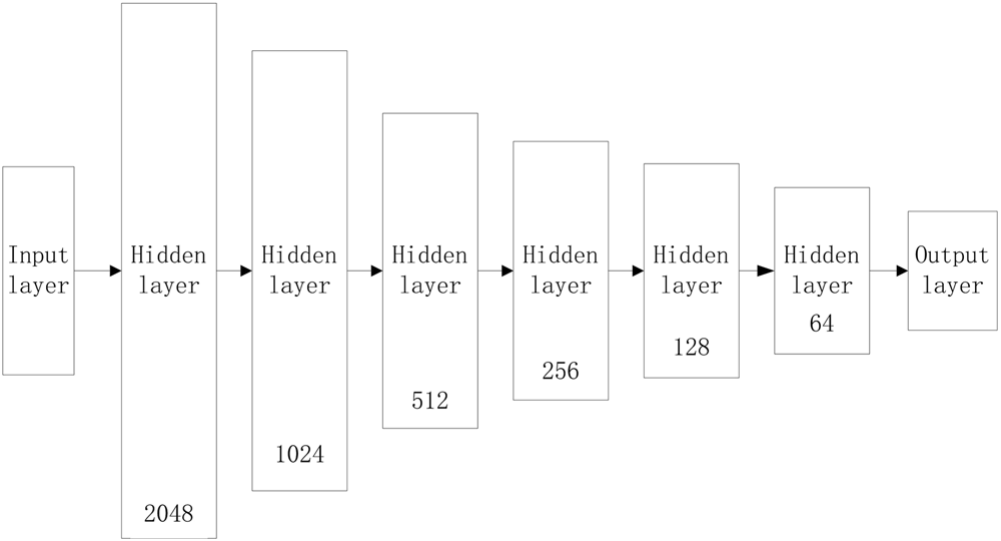
The framework of the DLM.

When analyzing the pure datasets, we use the deep learning model to make a binary classification as depicted in Fig. 2. However, for heterogeneous datasets, we can predict the samples by binary classification or multiple classification, respectively. To evaluate the prediction performance, we compare DPEH with MDR on prediction accuracy.

### Results on pure datasets

As mentioned above, the test samples in pure datasets will be classified as case or control. Consequently, the prediction can be considered as a binary classification.

**Figure 3:**
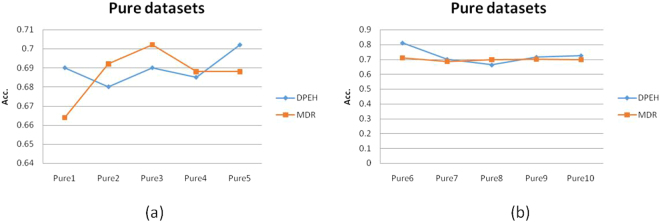
The prediction accuracy on pure datasets: (**a**) All the pure datasets are generated by 2 epistatic SNPs; (**b**) All the pure datasets are generated by 3 epistatic SNPs.

In the Fig. 3(a), both DPEH and MDR use 2 epistatic SNPs as features to build classification model and we find that the results of prediction accuracy are mostly around 69%. From these results, we cannot tell which method is dominant, which means that for pure datasets our method DPEH can serve as a possible alternative to MDR. We also find that during the training of DPEH, the accuracy of cross validation increases as the number of iterations grows. However, when predicting on test samples, the accuracy of cross validation is slightly higher than testing accuracy. In the Fig. 3(b), both DPEH and MDR use 3 epistatic SNPs as features to build classification model, our method DPEH is better than MDR. For Pure6, its accuracy reaches 81%.

### Results on heterogeneous datasets

In this study, heterogeneous datasets are simulated with two disease models H1 and H2. In the step of prediction, we can simply classify a test sample as normal or sick (binary classification), while we also can precisely predict the subtypes of sample (triple classification: normal, H1 or H2). Therefore, with using DPEH, researchers can choose to make a binary classification denoted as DPEH(2) or triple classification denoted as DPEH(3).

**Figure 4:**
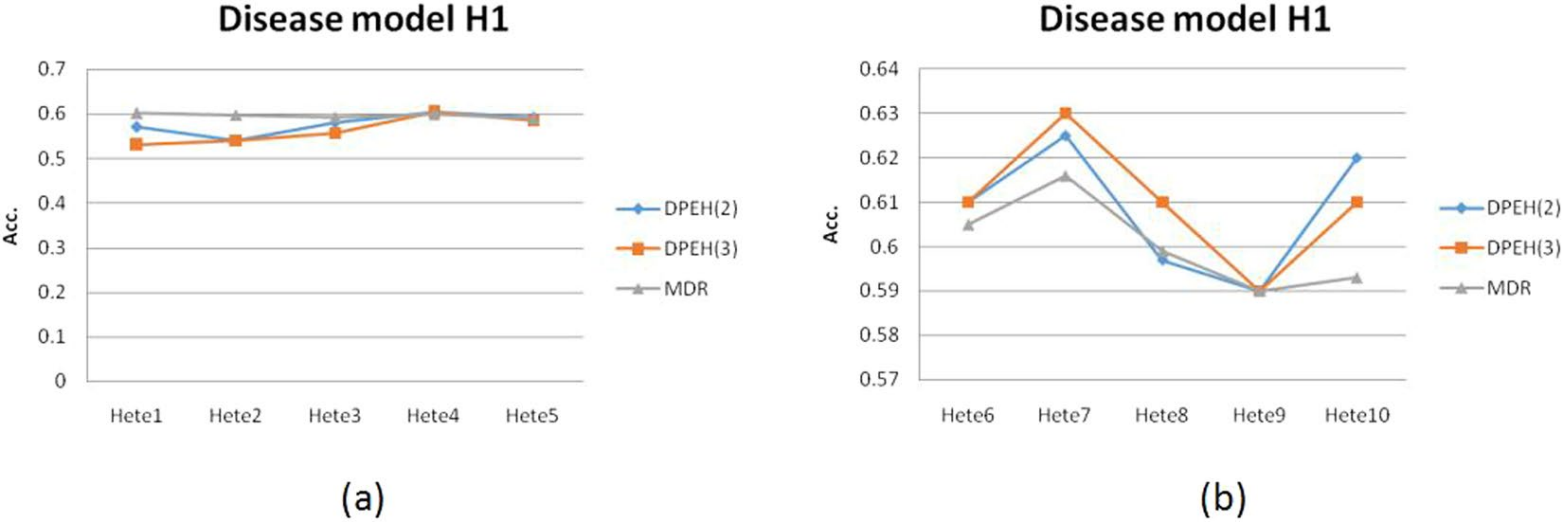
The prediction accuracy in disease model H1: (**a**) All the datasets are generated by 2 epistatic SNPs; (**b**) All the datasets are generated by 3 epistatic SNPs.

**Figure 5:**
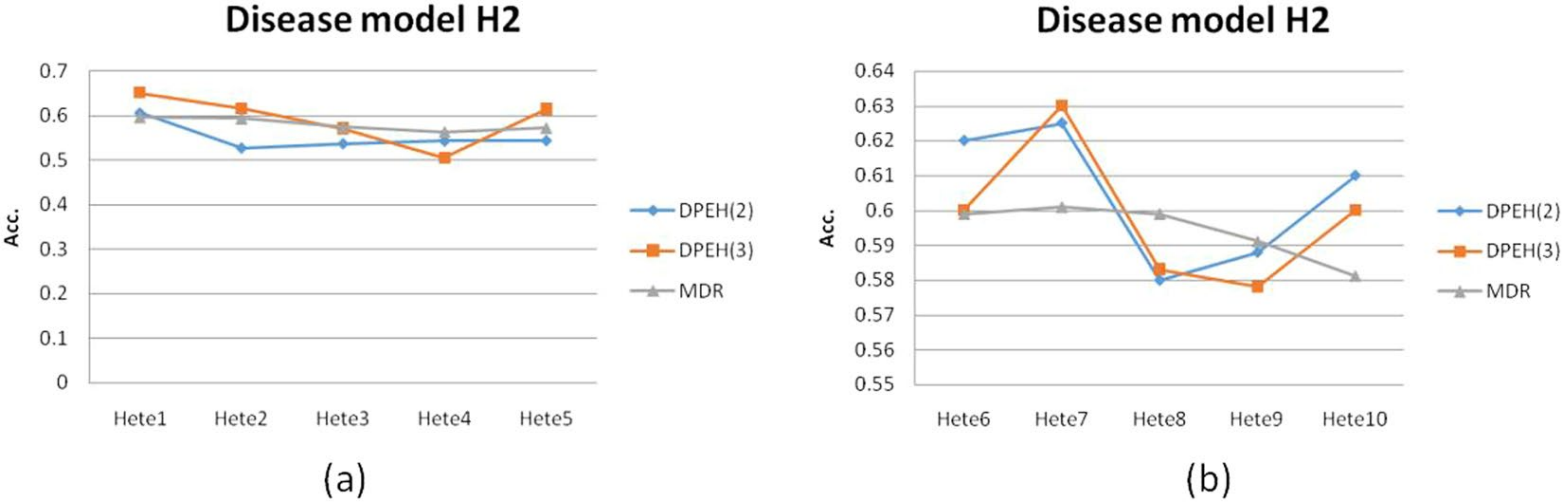
The prediction accuracy in disease model H2: (**a**) All the datasets are generated by 2 epistatic SNPs; (**b**) All the datasets are generated by 3 epistatic SNPs.

From Fig. 4(a), we find that MDR is slightly better than DPEH(2) and DPEH(3). However, for Fig. 4(b), it is interesting that DPEH is better than MDR, on average. We guess the reason is that the deep learning model may be more suitable for complex classification situations, especially when the sample size is large. But for simple situations or lack of training samples, the deep learning model may be underfitting, which is validated by Fig. 5(a,b).

**Figure 6:**
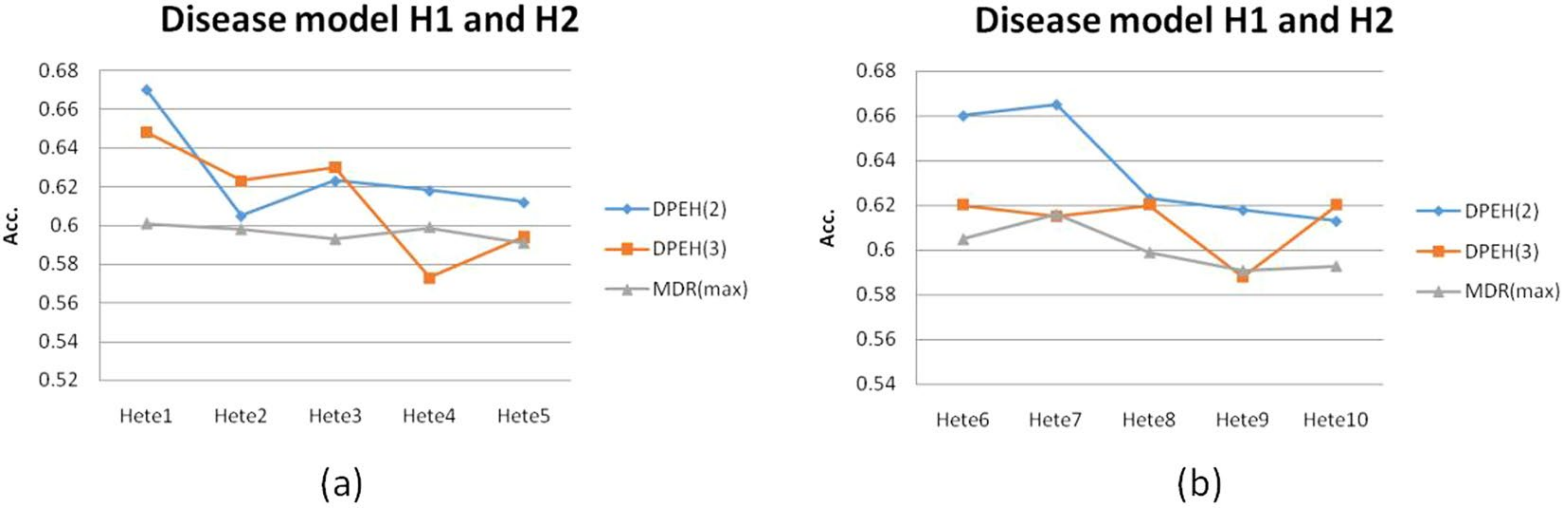
The prediction accuracy in disease model H1 and H2: (**a**) All the datasets composed with two disease models and each model involved in 2 epistatic SNPs; (**b**) All the datasets composed with two disease models and each model involved in 3 epistatic SNPs.

The comparison results of Fig. 6 demonstrate the most important merits of DPEH when handling heterogeneity. Because MDR cannot directly handle the heterogeneity, we select the maximum prediction accuracy value MDR(max) of H1 and H2 to represent the result of MDR.

From the results in Fig. 6, the DPEH shows better performance than MDR in most datasets. Note that both H1 and H2 compose with two epistatic SNPs in Fig. 6(a) but three epistatic SNPs in Fig. 6(b). If we use MDR to search epistasis with high order 4, MDR will return a best epistatic combination with prediction accuracy 0.67 on Hete1. But, none of the SNP in the best epistatic combination is the true pathogenic SNP. Therefore, MDR will experience a serious problem of generalization, which is not conducive for clinical research.

## Discussions

This paper has introduced a computational method DPEH which borrows a three-stage framework to concurrently handle epistasis and heterogeneity. Through the experimental results, we believe that DPEH has two main merits. First, DPEH returns several non-dominant epistatic combinations of SNPs which may contribute to different subtypes of complex diseases. It means that DPEH can effectively address the heterogeneity of complex disease. Furthermore, with using deep learning method, we can classify samples into more precise categories, namely subtypes of complex diseases. Consequently, DPEH may play important role in personal medical treatment. We now discuss some of the issues of DPEH:

### The scope of application

As mentioned above, the performance of DPEH is not always better than MDR, especially when the sample size is small or the epistatic pattern is simple (e.g. pure dataset and low epistatic order). In these simple situations, traditional machine learning models may show a comparable or even better performance. However, we find that as the sample sizes increases or the epistatic pattern becomes more complex, the merits of DPEH will become even more pronounced. Therefore, we suggest that when searching low epistatic order of epistasis in a small dataset, MDR may be a primary choice. If researchers need to handle heterogeneity or search high order epistasis, DPEH may be more suitable.

### Epistasis order

Theoretically, DPEH and MDR can search epistasis with order larger than 3. At the same time, when more SNPs are used as features, the prediction accuracy of training model may increase. However, only the 2 and 3 orders of epistasis are analyzed in this work. This is because in practical applications, the number of SNPs involved in epistasis is unlikely too big^2,15^. If we train prediction model with non-pathogenic biomarkers, the prediction accuracy on independent (test) samples will decrease significantly.

### The parameters of Clustering algorithm

In this study, only epistatic SNPs are considered as the features of samples, which means that the dimension of the data input to *K*-means clustering is equal to the epistatic order. For the number of clusters *K*, we can set the value of *K* to be the number of disease models. In this work, we simulate heterogeneous datasets with two disease models. In practical applications, the number of clusters always derives from prior knowledge of clinic research. It means that before clustering, we should search prior knowledge for a specific complex disease to determine the number of subtypes. Note that control samples do not need to be clustered and in the clustering step only case samples are clustered into different clusters corresponding to different subtypes.

### The implement of deep learning model

In the Fig. 2, the architecture of DLM is illustrated. The input dimension of the deep learning model is the number of SNPs in the datasets and output dimension is equal to the total number of classes. Apart from the input and output layer, there are millions of weights (2048*1024*512*256*128*64 = 2^51^) during the fully connected hidden layers. Therefore, mini-batch algorithm and GPU device are applied for effectively training. To avoid overfitting, the value of dropout is set to be 0.5. Nowadays, there are lots of successful frameworks for building deep learning model such as TensorFlow^16^, Caffe^17^ and so on. Keras written in Python is a high-level neural networks API, providing features such as user friendliness, modularity and easy extensibility. With using it, researchers can quickly build a prototype model to validate their ideas.

### Computing resources

Both DPEH and MDR have apply parallel computing techniques to accelerate the model training. Note that high-performance computing platform is important for practical applications, especially when the sample size of training data and the number of training epochs are large. In this work, we training our deep learning method on GTX 1080 (Total memory: 8.00GiB; MemoryClockRate 1.873 GHz). In addition, the offline training of DLM is also useful for improving the scalability and adaptability.

In summary, DPEH is an alternative to existing methods for epistasis analysis, with interesting characteristics. Among these, we demonstrate that DPEH can find complementary epistatic combinations contributing to different subtypes of complex diseases. Another advantage is that it is capable to recognize the subtypes of samples and help researches to carry on personal medical treatments.

Although DPEH is potentially beneficial for heterogeneity and epistasis analysis, several aspects should be addressed in further study. For example, for genome-wide epistasis analysis, the architecture of DPEH may be quite time consuming or even unsuitable, so that DPEH should be further investigated on real genomic data of complex disease. In addition, friendly graphic user interface should be developed for non-computer science professionals. Last but not least, in order to further improve the diagnostic accuracy of complex diseases, DPEH should fuse other biological information such as ncRNA biomarkers^18–28^.

## Methods

### Materials and evaluation

In this study, a widely used tool GAMETES_2.1^29^ can simulate both pure and heterogeneous datasets for evaluating DPEH. The GAMETES_2.1 is an easy-use software and provides parameters (e.g. minor allele frequency abbreviated as MAF, heterogeneity proportion, sample size, epistatic order and total number of SNPs) to customize various datasets. Table 1 lists the details of pure and heterogeneous datasets.

**Table 1:** The configurations of experimental datasets.

Data ID	Sample size	MAF	Heterogeneity proportion
Pure1	1000	(0.2, 0.2)	1.0
Pure2	2000	(0.2, 0.2)	1.0
Pure3	3000	(0.2, 0.2)	1.0
Pure4	4000	(0.2, 0.2)	1.0
Pure5	8000	(0.2, 0.2)	1.0
Pure6	1000	(0.2,0.2,0.2)	1.0
Pure7	2000	(0.2,0.2,0.2)	1.0
Pure8	3000	(0.2,0.2,0.2)	1.0
Pure9	4000	(0.2,0.2,0.2)	1.0
Pure10	8000	(0.2,0.2,0.2)	1.0
Hete1	1000	(0.2, 0.2) (0.3,0.3)	H1 = 50%, H2 = 50%
Hete2	2000	(0.2, 0.2) (0.3,0.3)	H1 = 50%, H2 = 50%
Hete3	3000	(0.2, 0.2) (0.3,0.3)	H1 = 50%, H2 = 50%
Hete4	4000	(0.2, 0.2) (0.3,0.3)	H1 = 50%, H2 = 50%
Hete5	8000	(0.2, 0.2) (0.3,0.3)	H1 = 50%, H2 = 50%
Hete6	1000	(0.2,0.2,0.2) (0.3,0.3,0.3)	H1 = 50%, H2 = 50%
Hete7	2000	(0.2,0.2,0.2) (0.3,0.3,0.3)	H1 = 50%, H2 = 50%
Hete8	3000	(0.2,0.2,0.2) (0.3,0.3,0.3)	H1 = 50%, H2 = 50%
Hete9	4000	(0.2,0.2,0.2) (0.3,0.3,0.3)	H1 = 50%, H2 = 50%
Hete10	8000	(0.2,0.2,0.2) (0.3,0.3,0.3)	H1 = 50%, H2 = 50%

All of these datasets contains 100 SNPs. The ‘Pure’ prefix of dataset ID denotes that the dataset is pure. The heterogeneity proportion of pure dataset equals to 1.0 and in pure dataset there is only one disease model. For two epistatic SNPs disease model, the MAFs are (0.2, 0.2). For three epistatic SNPs, their MAFs are (0.2, 0.2, 0.2). For heterogeneous datasets with ‘Hete’ prefix, there are two disease models (H1 and H2) coexisting and each disease model takes 50%. For dataset Hete1, the MAFs of H1 are (0.2, 0.2) and the MAFs of H2 are (0.3, 0.3). For dataset Hete6, the epistatic order is 3 and the MAFs of H1 are (0.2, 0.2, 0.2) and the MAFs of H2 are (0.3, 0.3, 0.3). Note that MAF can be set between 0 and 0.5, but it will result in a very large combination of parameters for simulating datasets. Therefore, we only selected representative values 0.2 and 0.3 for MAF.

For complex diseases diagnosis, prediction accuracy is a natural measure to evaluate the performance of proposed method. However, ‘high dimension but small sample’ could lead to overfitting, so that training accuracy, validation accuracy and test accuracy may be quite different. To fairly get the performance of DPEH, here we randomly select 10% samples from each dataset as test samples. Then, divide the remaining samples into 90% training samples and 10% validation samples. The accuracy is defined as equation (1).

1 $$Acc=\frac{n}{N}$$

where *N* is the total number of samples tested and *n* is the number of samples correctly classified.

### The framework of DPEH

Addressing epistasis and heterogeneity in a three-stage framework as illustrated in Fig. 1, DPEH firstly uses a epistasis detection step to search candidate epistatic combinations based on multi-objective optimization and then the Chi-square test is applied to filter false negative epistasis by statistical significance analysis. After that, for clustering stage, a *K*-means clustering algorithm is utilized to recognize potential subtypes. In this stage, we will use the clustering results to relabel the cases, dividing into multiple subtypes. Finally, we will use the deep learning method to predict the status (subtypes) of samples.

### The first stage: epistasis detection

To fully capture the heterogeneity existing in samples, we use ESMO to detect epistasis. For the completeness of the description, the relevant details of the ESMO are introduced.

By using equation (2), we can measure the information contribution of a *k* order epistatic combination to sample state *Y* (or vice versa).

2 $$I(Y|{X}_{1},\mathrm{..}.{X}_{k})=H(Y)+H({X}_{1},\mathrm{..}.{X}_{k})\,-\,H(Y,{X}_{1},\mathrm{..}.{X}_{k})$$

where *X* represents a SNP and I(*Y*|X_1_, … X_k_) denotes the uncertainty reduction of the sample state when the *k*-epistatic combination is observed.

The K2 score is defined as equation (3) when the prior distribution is assumed to be a Dirichlet distribution $D[{\alpha }_{11},\mathrm{..}.{\alpha }_{ij}]$. When there is no prior knowledge about pathogenesis, ${\alpha }_{{\rm{ij}}}=1$.

3 $$K2=\sum _{{\rm{i}}=1}^{I}(\sum _{b=1}^{{r}_{i}+1}\mathrm{log}(b)-\sum _{j=1}^{2}\sum _{d=1}^{{r}_{ij}}\mathrm{log}(d))$$

where *I* is the number of epistatic combinations and *I* = 3^k^. ${r}_{i}$ is the frequency of *i*-th genotype in all samples and ${r}_{ij}$ denotes the number of *i*-th genotype in samples with *j*-th state.

For Chi-square tests equation (4), suppose that *m* observations randomly drawn from a population are divided into *s* classes and in each class there are *O*_*i*_ samples.

4 $${\chi }^{2}=\sum _{i=1}^{s}\frac{{({O}_{i}-{E}_{i})}^{2}}{{E}_{i}}$$

where *E*_*i*_ is the expected number of *i*-th class.

### The second stage: clustering

After the epistasis detecting stage, multiple pathogenic genotype may pass the significance test. In this stage, we will use these epistatic SNPs as features to cluster all case samples. It means that all cases can be divided into several subtypes. Note that this stage is alternative. If the clustering stage is not applied, the prediction stage (the third stage) will run a binary classification. If this stage is implemented, the prediction can be taken as a multiple classification.

In this stage, lots of popular clustering algorithms can be applied to recognize the subtypes within the cases, such as density-based methods and hierarchical clustering method and so on. Various applications have proved that *K*-means clustering is a simple yet powerful tool^30^. In this study, we also apply the K-means to classify *m* samples into *K* subtypes S = {S_1_, S_2_, …, S_K_}(*K* ≧ 2). The dissimilarity between samples can be calculated by Euclidean distance on epistatic SNPs.

Since the cases are divided into several subtypes, we will relabel all the cases according to the cluster results. This process may play an important role in complex diseases diagnosis for personal medical treatment. Note that the value of *K* is determined by the prior knowledge of complex diseases.

### The third stage: prediction

Prediction is a key stage for building diagnosis model for complex diseases. Using deep learning model (DLM), it can not only elevate the performance of prediction, but also quicken the response by offline training. Deep learning methods use deep neural networks to portray the data in hierarchical abstractions, and they have been successfully applied in various studying area, such as image recognition^31^, speech recognition^32^ and so on. And, many studies believe that the deep learning model can help the bioinformatics researchers to make new breakthroughs^33,34^.

In the input layer of the DLM, the number of epistatic SNPs equals to the number of neurons. And, rectified activation function adopted in this study is defined as equation (5).

5 $$f(a)=\,\max (0,a)$$

In the output layer of the DLM, there are *C* nodes and *C* equals to the number of classes involved in prediction. The activation function of output nodes is a *softmax* function which is a generalization of the logistic function defined as equation (6).

6 $$\sigma {({\bf{z}})}_{j}=\frac{{e}^{{z}_{j}}}{{\sum }_{c=1}^{C}{e}^{{z}_{j}}}$$

where **z** is a *C*-dimensional vector and z_*j*_ is a real value in the range [0, 1].

To reduce overfitting in deep neural networks, we apply a regularization technique dropout which random drops out both hidden and visible units in neural network for preventing complicated co-adaptations on training data. Studies proved^35^ that it is a very simple way but efficient to prevent neural networks from overfitting.

Using the platform Keras (https://keras.io/), we build a deep neural network with 8 layers. And the infrastructure of our DLM is depicted as Fig. 2. The numbers of each hidden layer are the total number of neural nodes.

In Fig. 2, neurons in different layers are fully connected, so that there are lots of parameters that will be adjusted during training. In this work, to quicken the process of training, the mini-batch technique is used in model fitting. In addition, we also use GPU to accelerate the training based on a device GTX 1080.
